# Global trends in tertiary lymphoid structures: a bibliometric analysis from 2014 to 2023

**DOI:** 10.3389/fimmu.2024.1475062

**Published:** 2024-11-15

**Authors:** Yiwen Bao, Zeming Mo, Shuang Wang, Jinhua Long, Honghong Zhang, Yujun Xu, Honglian Jiang, Tianbao Qian, Zhu Zeng

**Affiliations:** ^1^ Key Laboratory of Microbio and Infectious Disease Prevention & Control in Guizhou Province, Key Laboratory of Infectious Immune and Antibody Engineering of Guizhou Province, Engineering Research Center of Cellular Immunotherapy of Guizhou Province, School of Basic Medical Sciences, Guizhou Medical University, Guiyang, China; ^2^ Immune Cells and Antibody Engineering Research Center of Guizhou Province, Key Laboratory of Biology and Medical Engineering, School of Biology and Engineering, Guizhou Medical University, Guiyang, China; ^3^ Department of Oncology, The Second Affiliated Hospital of Zunyi Medical University, Zunyi, China; ^4^ Department of Head & Neck, Affiliated Tumor Hospital of Guizhou Medical University, Guiyang, China; ^5^ Department of Nephrology, The People’s Hospital of Qiannan, Duyun, Guizhou, China

**Keywords:** bibliometric, immunotherapy, tertiary lymphoid structures, research trend, prognostic value

## Abstract

**Aim and background:**

Tertiary lymphoid structures (TLS) are increasingly recognized for their role in immunity. Despite growing interest, a systematic bibliometric analysis of TLS-related research has been lacking. To provide a comprehensive overview of current research trends and hotspots, we conducted a bibliometric analysis using data from the Web of Science Core Collection.

**Methods:**

We retrieved TLS-related publications from the Science Citation Index Expanded within the Web of Science Core Collection from January 2014 to December 2023. Co-occurrence analysis with “VOSviewer” identified current status and research hotspots, while “CiteSpace” was used for co-citation analysis to assess knowledge evolution and bursts. Thematic evolution was explored using bibliometrics to identify emerging keyword trends. Additionally, we examined country/region, institutional, and author contributions and collaborations. Tables were created using Microsoft Word.

**Results:**

A total of 785 publications were analyzed, showing a continuous growth trend from 2017 to 2023, indicating escalating interest in TLS among researchers. Leading countries in TLS research were China (231 publications), the United States (212 publications), and France (89 publications). The most productive institution and author were the “Institut national de la santé et de la recherche médicale” (70 publications) and Catherine Sautes-Fridman (21 publications), respectively. Key topics included TLS, B cells, and immunotherapy. Recent research has focused on mechanisms linking TLS with cancers, such as immunotherapy, tumor microenvironment, tumor-infiltrating lymphocytes, prognosis, and immune checkpoint inhibitors, highlighting an expanding area of study. Additionally, TLS’ potential as a biomarker for predicting immunotherapy efficacy across different cancer types remains a burgeoning research direction.

**Conclusions:**

This study provides a comprehensive analysis of global TLS-related publications, revealing key literature metrics and identifying influential articles and emerging research concerns. These findings contribute valuable insights into the role of TLS in immunotherapy and suggest future directions for this dynamic field.

## 1 Introduction

Immune cells are frequently found in the microenvironment surrounding tumor cells (1). The prognostic impact of these tumor-infiltrating immune cells across different cancer types has long been of interest (2–4). In recent years, immunotherapy, particularly the use of immune checkpoint inhibitors (ICIs), has made remarkable strides in antitumor therapy (5). ICIs primarily enhance antitumor efficacy by reversing the functional exhaustion of lymphocytes infiltrating within or around tumors (6, 7). Consequently, there has been increased attention on the prognostic role and regulatory mechanisms of these tumor-infiltrating immune cells. For instance, patients with enriched intratumoral immune cell populations in non-small cell lung cancer (NSCLC) treated with ICIs often exhibit higher response rates and improved outcomes (8, 9). In chronic inflammatory or tumor environments, a type of lymphocyte aggregate known as tertiary lymphoid structures (TLS) frequently forms (10). TLS develops postnatally in non-lymphoid tissues, also referred to as ectopic lymphoid structures. TLS has been identified in autoimmune diseases and chronic infections (11, 12). Additionally, several studies suggest that the presence of TLS in tumor tissue serves as a promising prognostic marker for immunotherapy (9, 13, 14). However, TLS may not consistently predict positive outcomes in certain tumor subtypes (15, 16). This variability in immunotherapy outcomes has further stimulated research into the functions and regulatory mechanisms of TLS within tumors.

The structure, cellular composition, and regulation of TLS may be different between diseases (10). As a result, investigating the mechanisms of action and the potential therapeutic predictive role of TLS has emerged as a prominent research area, leading to the publication of numerous related studies. Bibliometric analysis, a statistical methodology utilizing public literature databases, quantitatively and qualitatively assesses relevant publications to summarize research trends and hotspots in a specific field (17). Widely applied in scientific research, bibliometrics systematically analyzes various aspects of a research field, including countries/regions, keywords, references, authors, journals, and institutions (18, 19). However, the comprehensive exploration of global trends in TLS through bibliometric analysis remains relatively unexplored.

Therefore, conducting a bibliometric analysis to explore research trends and hotspots related to TLS can significantly aid researchers in quickly grasping essential information, identifying pivotal developments, and discerning future directions in TLS research.

## 2 Materials and methods

### 2.1 Data collection and extraction

Data from the Web of Science Core Collection database (WOSCC) were utilized for this study. Articles and reviews in English were retrieved from the WOSCC on February 27, 2024. The search terms in this article are derived from the subject terms in the MeSH database (https://www.ncbi.nlm.nih.gov/mesh). We defined the search keywords as: “Tertiary lymphoid structure*”, “Lymphoid Structure*, Tertiary”, “Ectopic Lymphoid-Like Structure*”, “Lymphoid-Like Structure*, Ectopic”, “Lymphoid Formation*, Ectopic”, “Ectopic Lymphoid Tissue*”, “Lymphoid Tissue*, Ectopic”, “Ectopic Lymphoid Organ*”, “Lymphoid Organ*, Ectopic”, “Ectopic Lymph Node*”, “Lymph Node*, Ectopic”, “Ectopic Lymphoid Follicle*”, and “Lymphoid Follicle*, Ectopic”.

**Figure 1:**
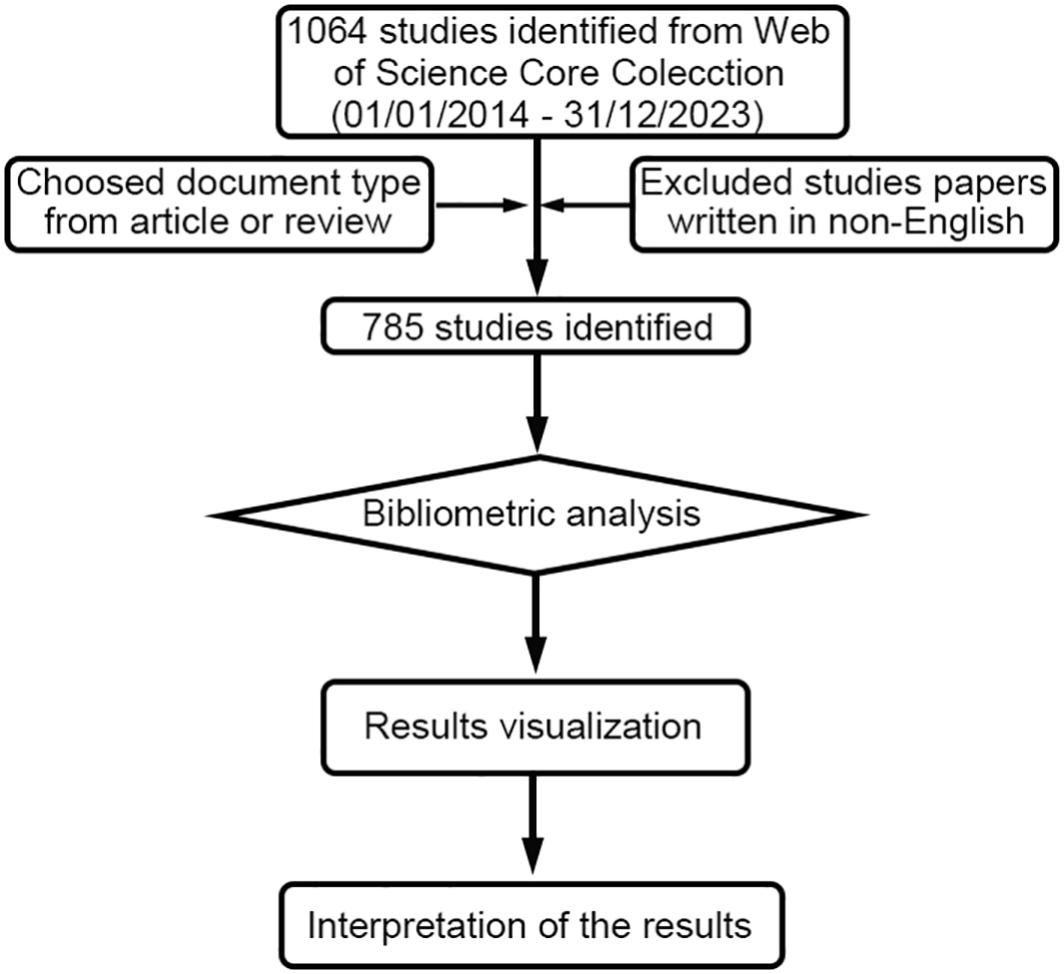
The diagram illustrates the process of data filtering and bibliometric analysis.

The terms attached to TLS were searched in the titles (TI), abstracts (AB), or author keywords (AK), a search strategy commonly used in bibliometric analysis (20–22). The detailed search formula is as follows: ((TI=(“Tertiary lymphoid structure*” OR “Lymphoid Structure*, Tertiary” OR “Ectopic Lymphoid-Like Structure*” OR “Lymphoid-Like Structure*,Ectopic” OR “Lymphoid Formation*, Ectopic” OR “Ectopic Lymphoid Tissue*” OR “Lymphoid Tissue*, Ectopic” OR “Ectopic Lymphoid Organ*” OR “Lymphoid Organ*, Ectopic” OR “Ectopic Lymph Node*” OR “Lymph Node*, Ectopic” OR “Ectopic Lymphoid Follicle*” OR “Lymphoid Follicle*, Ectopic”)) OR AB=(“Tertiary lymphoid structure*” OR “Lymphoid Structure*, Tertiary” OR “Ectopic Lymphoid-Like Structure*” OR “Lymphoid-Like Structure*,Ectopic” OR “Lymphoid Formation*, Ectopic” OR “Ectopic Lymphoid Tissue*” OR “Lymphoid Tissue*, Ectopic” OR “Ectopic Lymphoid Organ*” OR “Lymphoid Organ*, Ectopic” OR “Ectopic Lymph Node*” OR “Lymph Node*, Ectopic” OR “Ectopic Lymphoid Follicle*” OR “Lymphoid Follicle*, Ectopic”)) OR AK=(“Tertiary lymphoid structure*” OR “Lymphoid Structure*, Tertiary” OR “Ectopic Lymphoid-Like Structure*” OR “Lymphoid-Like Structure*,Ectopic” OR “Lymphoid Formation*, Ectopic” OR “Ectopic Lymphoid Tissue*” OR “Lymphoid Tissue*, Ectopic” OR “Ectopic Lymphoid Organ*” OR “Lymphoid Organ*, Ectopic” OR “Ectopic Lymph Node*” OR “Lymph Node*, Ectopic” OR “Ectopic Lymphoid Follicle*” OR “Lymphoid Follicle*, Ectopic”). Our selection criteria included articles published in English between 1 January 2014 and 31 December 2023. The process of article selection and review is illustrated in **Figure 1**.

### 2.2 Data analysis and visualization

The study was conducted by two independent researchers to ensure the reliability of the results. These researchers evaluated the outcomes of independent scans and included studies that met the predefined inclusion and exclusion criteria. Documents retrieved from the search were downloaded in plain text format, and relevant information including author details, country or region, institution, keywords, and references was extracted for subsequent data analysis. In our analysis, we examined various aspects including journals, countries, institutions, cited publications, authors, keyword co-occurrence networks, abstract content analysis, and co-citations. We utilized several software tools for this purpose: “VOSviewer 1.6.17”, “CiteSpace 5.8 R3”, and the “bibliometrics” function in R Version 4.2.2 (R Foundation for Statistical Computing, Vienna, Austria; http://www.R-project.org/). Additionally, Tables were created using Microsoft Word.

## 3 Results

### 3.1 Annual publications and trends

**Figure 2:**
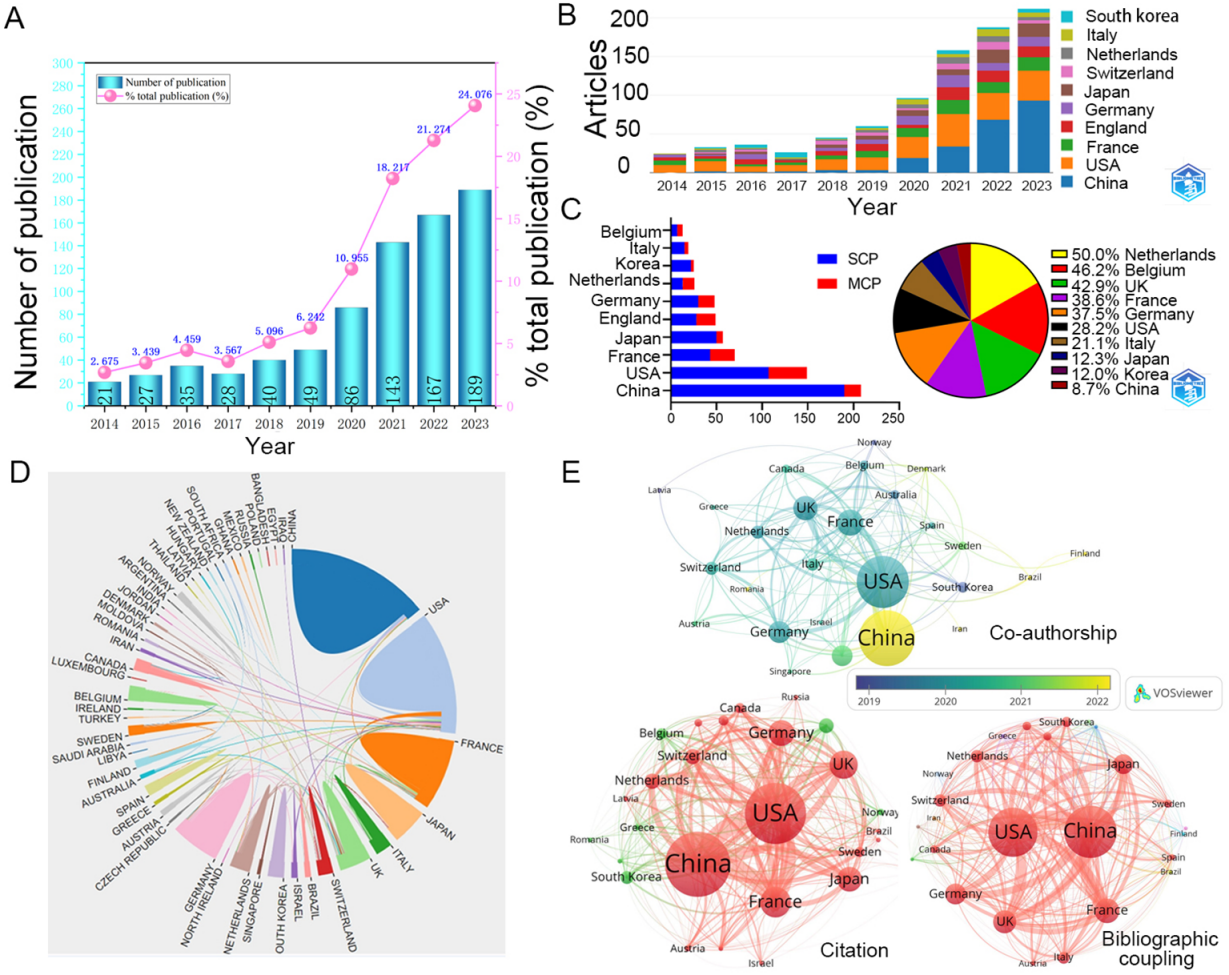
Annual publications and interactions between countries. **(A)** The total number and growth rate of TLS-related publications per year during 2014-2023. **(B)** Comparison of the number of articles published in several countries over 10 years. **(C)** The partnerships of different countries. **(D)** Visual map of transnational/regional cooperation. The size of the border lines separating countries indicates the extent of cooperative interaction. **(E)** The networks of cooperation between 53 countries with at least one publication. SCP, Single country publications; MCP, Multiple Country publications; USA, the United States of America; UK, the United Kingdom.

A total of 589 articles (74.8%) and 198 reviews (25.2%) related to TLS were included in the WOSCC. Of these, 785 publications were written in English. Over the period from 2014 to 2023, there has been a consistent annual increase in publications on TLS (**Figure 2A**). The growth rate reached its peak in 2023 at 24.076%, indicating a significant rise in interest in TLS within the academic community.

Since 2014, articles originating in the United States (212 documents) have maintained a high percentage, China (231 documents) has seen a dramatic increase in the number of articles published in recent years and was expected to overtake the United States as the most published country after 2022 (**Figure 2B**). In addition, France, the United Kingdom, Germany, and Japan are also common countries for publication on TLS (**Table 1**). Single country publications are generally more numerous than multiple country publications. The countries with a high proportion of multiple country publications are mainly the Netherlands, Belgium and the United Kingdom, whereas China has the lowest proportion (**Figure 2C**). These collaborations have resulted in stronger linkages within the TLS research community in these countries compared to others (**Figures 2D, E**; **Table 1**). East Asian countries such as China, South Korea, and Japan have increasingly engaged in joint publications with authors from various nations. Additionally, countries like Brazil, Ireland, and Iran have also begun contributing notable results on TLS in recent years (**Figure 2E**). These trends underscore the global and collaborative nature of TLS research.

**Table 1:** The top 10 highly documents countries/regions for TLS research.

Country	Documents	Citations	Total link strength
China	231	3921	58
USA	212	11425	173
France	89	7655	84
UK	80	3834	102
Germany	71	3352	72
Japan	67	1722	35
Netherlands	37	3525	55
Switzerland	37	2621	52
Italy	35	941	42
South Korea	29	859	20

USA, the United States of America; UK, United Kingdom.

### 3.2 Analysis of keywords

#### 3.2.1 Gene signature

**Figure 3:**
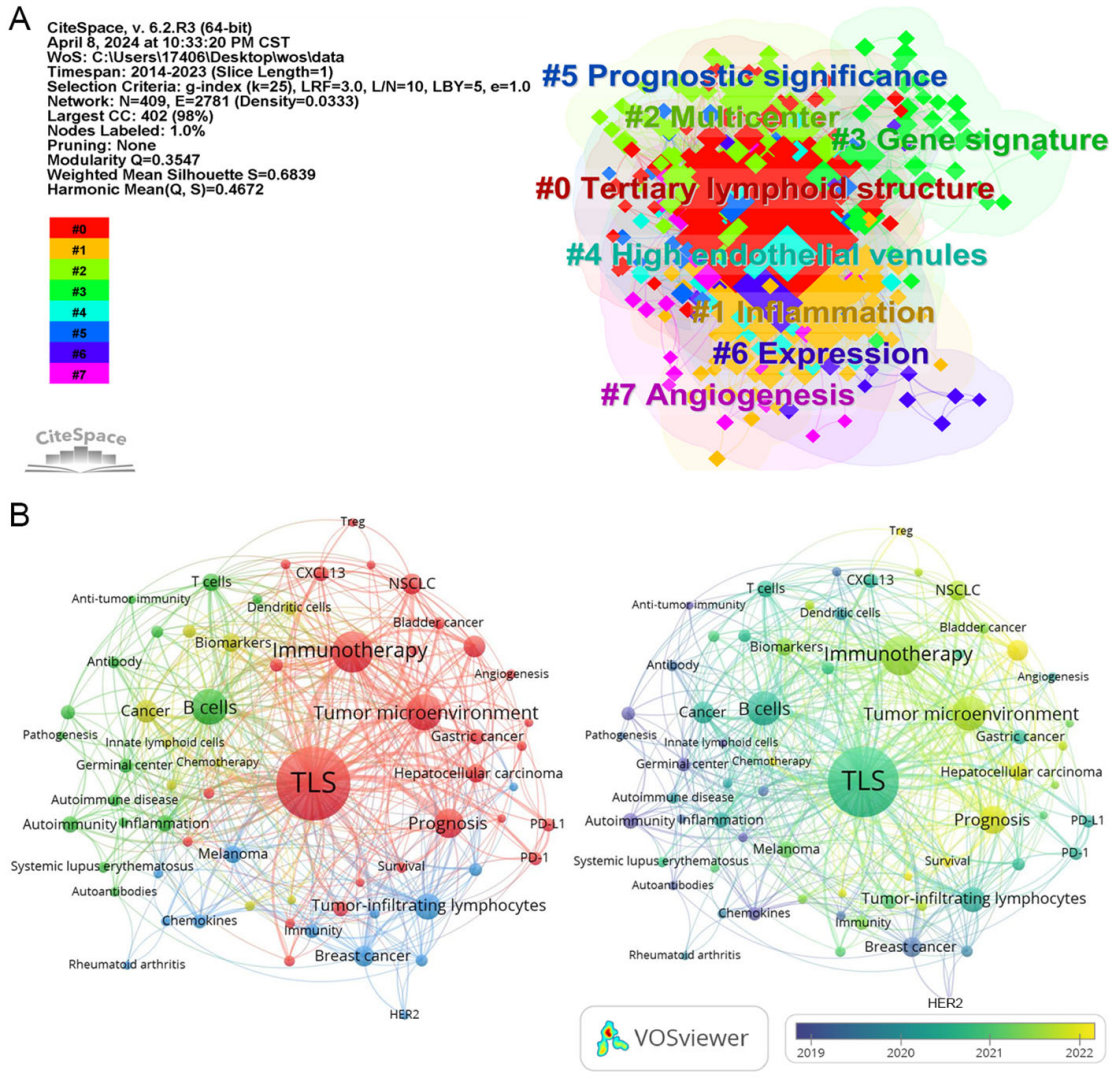
The network map of keywords. **(A)** the clustered network map of keywords in the field of TLS. **(B)** Network visualization of keyword co-occurrence which appeared at least 5 times (67 author keywords).

This keyword clustering map generated by “CiteSpace” reveals several primary research themes identified through keyword clustering in the literature. These themes include gene signature, prognostic significance, inflammation, high endothelial venules (HEVs), and angiogenesis (**Figure 3A**).

It is interesting to find suitable gene set scores to identify TLS. Of particular interest is the development of gene set scores tailored for identifying TLS. For instance, researchers have utilized a 12-gene expression signature (including CCL2, CCL3, CCL4, CCL5, and others) to assess TLS presence across different cancer types (23, 24). Other gene set scores were also attempted to be applied (15, 25). Given the variability and dynamics in gene expression, immunohistochemistry and hematoxylin-eosin staining continue to be crucial for determining TLS presence and abundance (23, 26). Thus, integrating pathological evaluation with gene signatures may offer a more comprehensive and accurate approach.

#### 3.2.2 Prognostic significance

The prognostic role of TLS in cancer treatment, particularly in immunotherapy, has garnered significant attention. Studies indicate that high intratumor TLS abundance in colorectal cancer, NSCLC, head and neck squamous cell carcinoma, hepatocellular carcinoma, and cutaneous melanoma is associated with improved patient survival and enhanced immunotherapy efficacy (23, 26–30).

However, research also reveals nuances where certain TLS subtypes may not uniformly predict favorable outcomes across all cancer types. For instance, high infiltration (>151) of scattered tumor-infiltrating lymphocytes is identified as a poor prognostic factor in metastatic colorectal cancer (31). Additionally, the level of TLS infiltration did not impact the prognosis of patients with acral melanoma receiving adjuvant anti-PD-1 therapy (15). Moreover, the presence of tumor-distal TLS correlates with poor prognosis in clear cell renal cell carcinoma, whereas tumor-proximal TLS exhibits the opposite effect (32).

These findings underscore the varied predictive value of TLS across different cancer types and even within the same cancer type based on factors such as anatomical location, structure, and abundance of TLS. These factors may influence the tumor immune microenvironment, ultimately impacting patient survival outcomes.

#### 3.2.3 HEVs and angiogenesis

Tumor-associated HEVs play a crucial role in facilitating efficient lymphocyte infiltration into tumors (33). They are also recognized as components of TLS (34, 35). Studies have highlighted that a dense presence of HEVs within tumors correlates with improved efficacy of immunotherapy, chemotherapy, and other treatments (33, 34, 36–38). However, elevated expression of immune checkpoint ligands on tumor-associated HEVs can hinder CD8-positive T cell infiltration, potentially leading to poorer prognosis in patients with NSCLC (39). Conversely, the presence of HEVs indicates effective treatment response in NSCLC patients receiving PD-1 inhibitors combined with anti-angiogenic therapy (40).

The relationship between angiogenesis and TLS is also noteworthy. For instance, intratumoral injection of the STING agonist ADU S-100 in melanoma induced vascular normalization and TLS formation, enhancing control over tumor growth (41). Similar observations have been made in pancreatic neuroendocrine tumors, where vascular normalization and TLS formation attenuated resistance to immunotherapy (42). These findings underscore the critical role of angiogenesis normalization and effective infiltration of antitumorigenic lymphocytes in reshaping the tumor immune microenvironment.

#### 3.2.4 Networks

In the keyword network analysis, TLS, B cells, immunotherapy, survival, and cancer emerge as the most frequently occurring keywords (**Figure 3B**; **Table 2**), highlighting TLS as a prominent area of research interest. Over time, additional keywords such as Treg, lung adenocarcinoma, chemotherapy, radiomics, and immune infiltration are gaining prominence in relation to TLS.

**Table 2:** The top 10 keywords for TLS research.

Rank	Count	Keywords	Rank	Centrality	Keywords
1	363	Tertiary lymphoid structures	1	0.1	Activation
2	292	B cells	2	0.09	Neogenesis
3	203	Immunotherapy	3	0.08	Expression
4	187	Survival	4	0.08	Germinal centers
5	187	Cancer	5	0.08	Receptor
6	159	Expression	6	0.07	Breast cancer
7	136	T cells	7	0.07	Antibody
8	117	Dendritic cells	8	0.07	Rheumatoid arthritis
9	109	Tumor-infiltrating lymphocytes	9	0.06	T cells
10	74	Tumor microenvironment	10	0.06	Tissue

For instance, CT-based radiomics nomograms have shown promise in predicting TLS presence in intrahepatic cholangiocarcinoma, while MRI radiomics appears to offer similar capabilities (43, 44). Radiomics’ noninvasive approach offers convenience for detecting TLS (43, 45). However, additional clinical evidence is required to validate the reliability of radiomics findings. Notably, “activation” emerged as the keyword with the highest centrality value in **Table 2**, highlighting a predominant focus on immune cell activation in TLS research.

#### 3.2.5 Citation bursts

**Figure 4:**
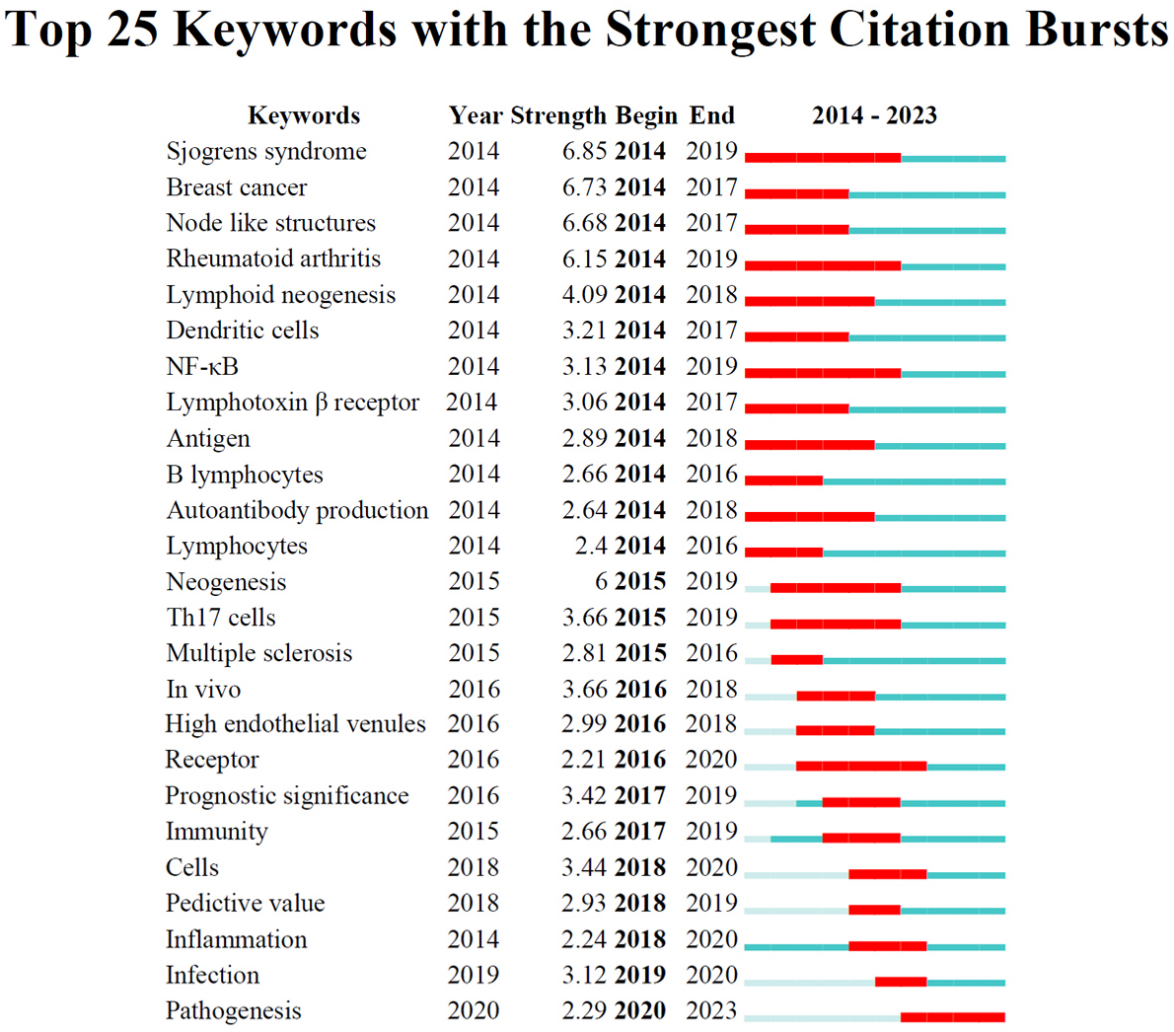
The top 25 keywords with the strongest citation bursts on TLS field.

To track the evolving research hotspots in TLS, we analyzed the top 25 keywords with the strongest citation bursts (**Figure 4**), organized by the onset year of their burst. From 2014 to 2018, significant areas of focus included “Breast cancer”, “Sjogrens syndrome”, “Node like structures”, “Rheumatoid arthritis”, “Lymphoid neogenesis”, “Dendritic cells”, “NF-κB”, “Lymphotoxin beta receptor”, “Antigen”, “B lymphocytes”, and “Autoantibody production”. Recent years have seen a shift towards keywords like “Cells”, “Predictive value”, “Inflammation”, “Infection”, and “Pathogenesis”.

When sorted by the duration of their burst, the “Sjogrens syndrome”, “Rheumatoid arthritis”, and “NF-κB “ exhibited the longest-lasting impact over a continuous three-year period. In terms of burst strength, which reflects significance, the “Sjogrens syndrome” peaked with a substantial citation spike of 6.85 intensity from 2014 to 2019, while “Pathogenesis” showed a notable spike of 2.29 intensity in 2020. These findings indicate that while early research likely explored the connection between TLS and autoimmune diseases (46, 47), recent trends suggest a growing interest in tumor immunity (12).

### 3.3 Analysis of references

**Figure 5:**
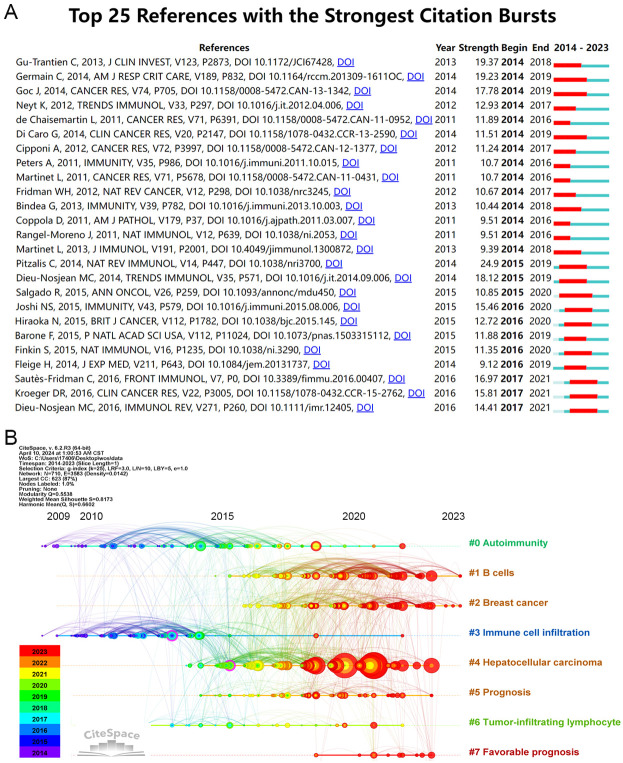
Analysis of literature references. **(A)** Top 25 references with the strongest citation bursts (sorted by the beginning year of burst). **(B)** The Timeline of co-cited literature related to TLS.

Burst-detection analysis identifies papers experiencing significant citation surges, pivotal for tracing the evolution of research domains. In the TLS field from 2014 to 2023, notable references were determined (**Figure 5A**). A standout publication is the 2013 study by Gu-Trantien C et al., “CD4^+^ follicular helper T cell infiltration predicts breast cancer survival” which exhibited the strongest citation burst (48). This study highlights that CD4^+^ follicular helper T cells within tertiary lymphoid structure germinal centers can predict survival and response to neoadjuvant chemotherapy in breast cancer. Moreover, it may be the first to identify follicular helper T cells as a subset of tumor-infiltrating T cells in solid tumors.

Another significant article, “Presence of B Cells in Tertiary Lymphoid Structures Is Associated with Protective Immunity in Patients with Lung Cancer” by Germain C et al. in 2014, ranks second in strength value (49). This research demonstrates that a high density of follicular B cells correlates positively with long-term survival in both early and advanced chemotherapy-treated NSCLC patients. Additionally, patients with high densities of intratumoral follicular B cells and mature dendritic cells exhibited significantly better survival outcomes. Furthermore, the article “Tertiary Lymphoid Structures in Cancers: Prognostic Value, Regulation, and Manipulation for Therapeutic Intervention” by Sautès-Fridman C et al., published recently, holds the highest strength value (16.97) (50). These findings underscore the integral role of TLS formation and maturity in tumor progression and response to treatment interventions.

The timeline analysis reveals 710 keywords grouped into 9 large clusters (see **Figure 5B**), indicating the evolution of co-cited literature by keyword clustering. Recent prominence is observed with keywords like “favorable prognosis” and “sarcoma,” suggesting active research engagement in TLS related to these areas. Concurrently, research on “autoimmunity” and “immune cell infiltration” has demonstrated sustained interest over time.

Notably, there has been a notable surge in TLS research related to hepatocellular carcinoma in recent years, coinciding with advancements in ICIs for HCC treatment (51). Recent studies highlight TLS as a reliable predictor of ICIs efficacy and prognosis (52, 53). This trend suggests that the predictive role of TLS in new cancer types will likely garner increased attention amid the expanding landscape of immunotherapy.

Analysis of references underscores the dynamic change of TLS research, reflecting both emerging trends and enduring themes in understanding their impact across different cancers and immunotherapeutic strategies.

### 3.4 Top contributing authors and institutions

**Figure 6:**
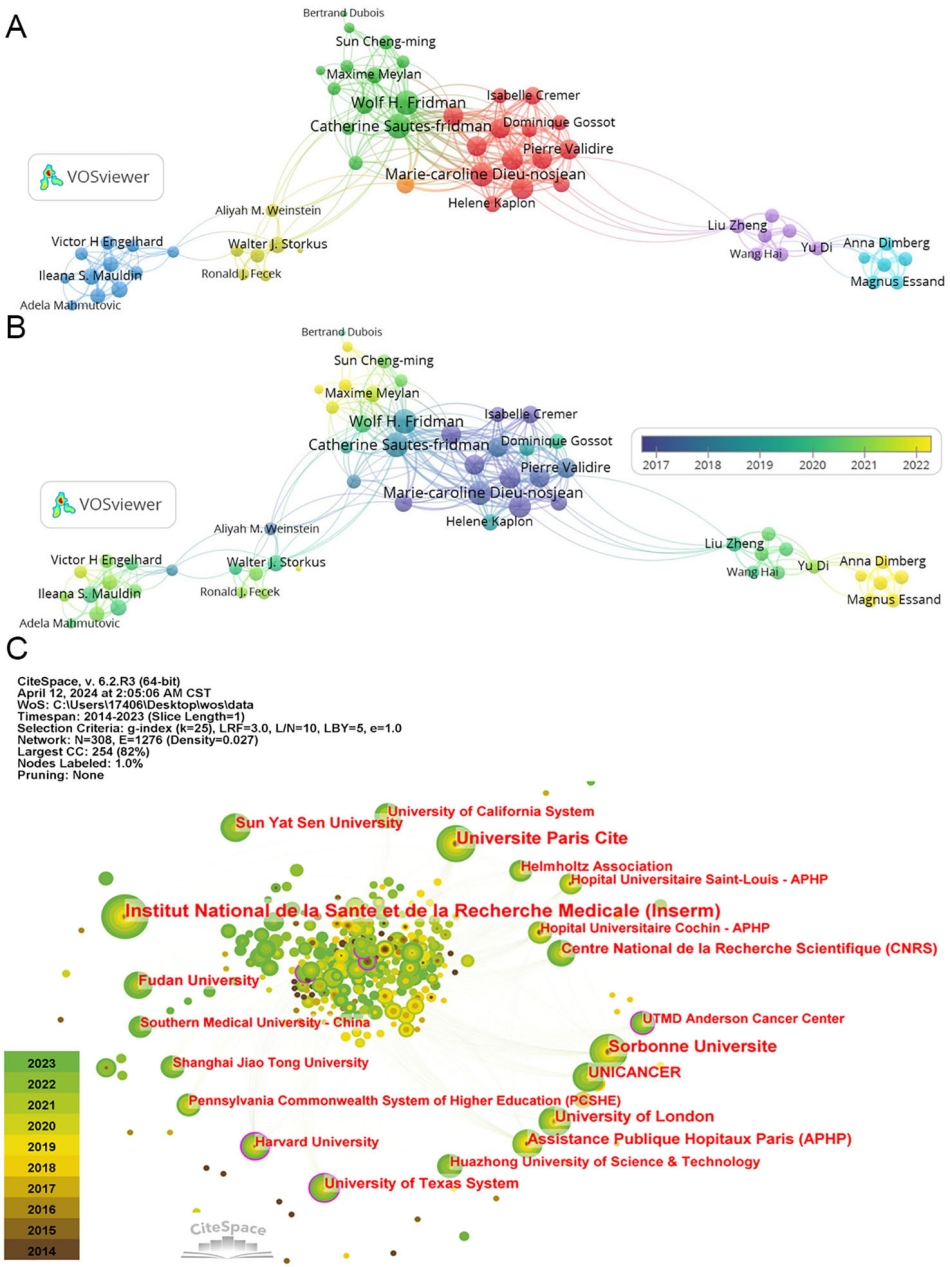
The co-authorship of authors and institutions. **(A)** The co-authorship network clusters of authors. **(B)** The co-authorship network timeline of authors. **(C)** The network of institutions.

A co-authorship network analysis utilizing “VOSviewer” software illustrates collaborative efforts among authors in TLS research spanning from 2014 to 2024. Among 5,739 authors, 224 have contributed three or more publications, resulting in the identification of 6 major network clusters (**Figure 6A**).

The top three authors by publication count are Catherine Sautes-Fridman (21), Wolf Herman Fridman (19), and Dieu-Nosjean Marie-Caroline (16) (**Table 3**), all affiliated with French research organizations. Their collaborative efforts have yielded numerous impactful articles in the TLS field (49, 54, 55). Similarly, Soizic Garaud, Denis Larsimont, and Karen Willard-Gallo hold the highest total link strength values (108), representing significant collaborative networks within Belgian research institutions (56, 57). In recent years, Alessandra Vaccaro and Anna Dimberg from Sweden, among others, have made notable contributions with multiple publications on glioma-associated TLS (**Figure 6B**) (58, 59). While these authors are highlighted, it’s important to acknowledge the diverse contributions of others who have propelled TLS research forward.

**Table 3:** The 18 most productive authors for TLS research.

Author	Documents	Citations	Total link strength	Author	Documents	Citations	Total link strength
Catherine Sautes-Fridman	21	3744	104	Denis Larsimont	10	879	108
Wolf-H. Fridman	19	3605	100	Walter J. Storkus	10	412	27
Dieu-Nosjean Marie-Caroline	16	2416	85	Karen Willard-Gallo	10	879	108
Michele Bombardieri	11	911	24	Francesca Barone	9	413	34
Soizic Garaud	11	882	108	Alexandre De Wind	9	866	100
Claire Germain	11	1674	72	In Ah Park	9	521	56
Gyungyub Gong	11	550	64	In Hye Song	9	910	18
Hee Jin Lee	11	550	64	Costantino Pitzalis	9	768	25
Craig L. Slingluff	11	330	35	Karina Silina	9	521	56

In terms of institutional contributions, node sizes denote the number of publications, while centrality reflects the influence of links passing through each node. Notably, TLS has garnered substantial attention across numerous research institutions. The top five institutions by publication volume include “Institut National de la Sante et de la Recherche Medicale” (70), “Universite Paris Cite” (49), “Sorbonne Universite” (45), “University of London” (33), and “UNICANCER” (30) (**Figure 6C**; **Table 4**). Recent years have seen increasing focus from Chinese research organizations like “Fudan University” (24) and “Sun Yat-sen University” (28), underscoring their emerging role in TLS research.

**Table 4:** Top 10 most productive institutions for TLS research.

Rank	Institutions	Count	Centrality	Year
1	Institut National de la Sante et de la Recherche Medicale (Inserm)	70	0.07	2014
2	Universite Paris Cite	49	0.05	2014
3	Sorbonne Universite	45	0.05	2014
4	University of London	33	0.07	2014
5	UNICANCER	30	0.01	2018
6	Assistance Publique Hopitaux Paris (APHP)	29	0.07	2014
7	Sun Yat Sen University	28	0.01	2020
8	University of Texas System	24	0.14	2017
9	Fudan University	24	0.02	2020
10	Centre National de la Recherche Scientifique (CNRS)	24	0.07	2018

Moreover, institutions like “Harvard University” and “the University of Texas System”, marked with a purple ring denoting high centrality nodes, serve as pivotal connectors in the scholarly communication and collaboration network within this domain. Maybe their influence extends across various research teams, facilitating interdisciplinary advancements in TLS research.

### 3.5 Distribution of the top cited journals

**Figure 7:**
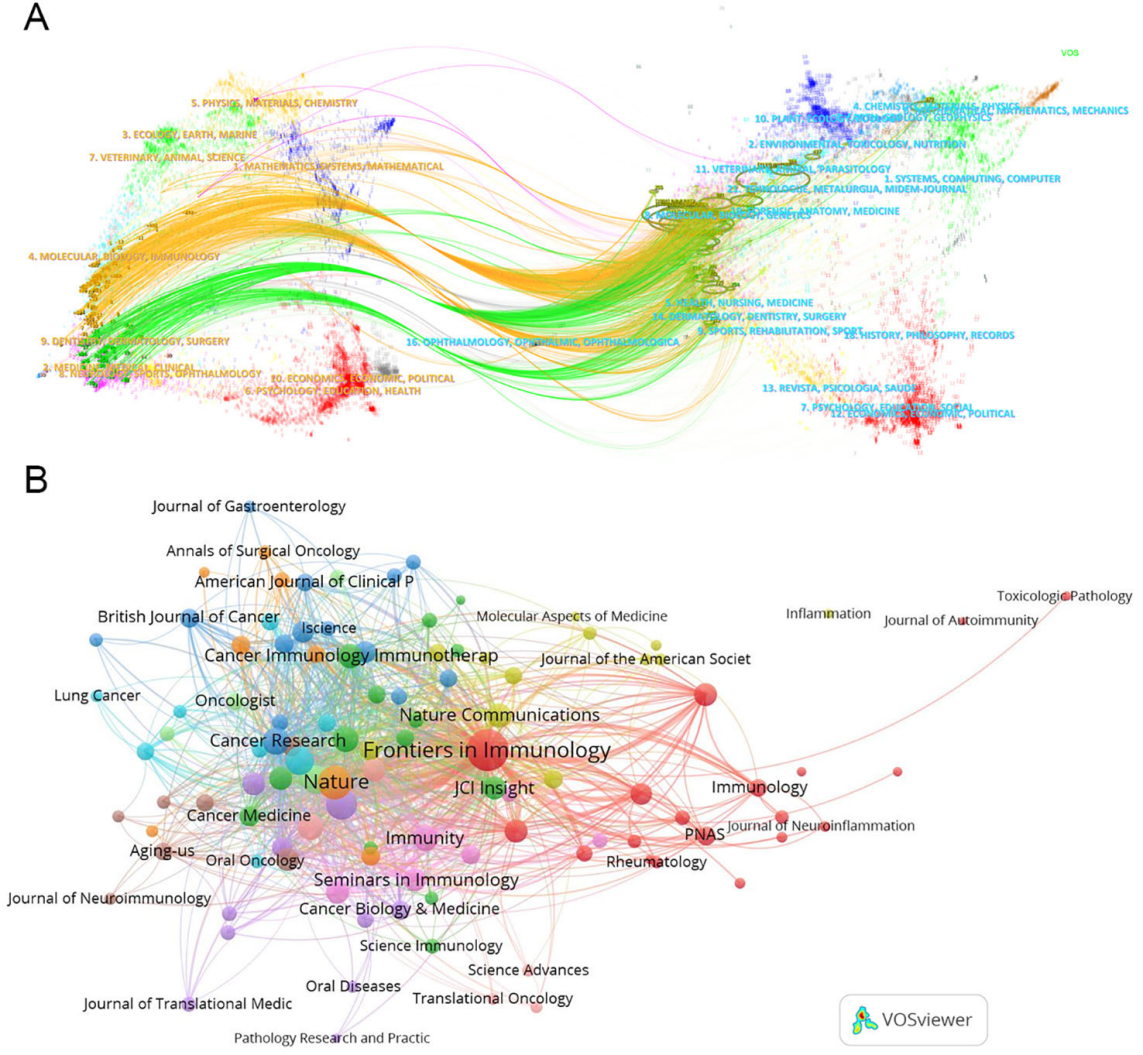
Collaboration among journals. **(A)** The dual map overlay of journals. This map uses different colors to symbolize the journals in different disciplines. The map can be divided into two parts, the left side is the distribution of the cited journals, which represents the main disciplines of Science mapping, and the right side is the distribution of the cited journals. **(B)** The network map of cited journals.

Using “CiteSpace” software, a dual map overlay of journals reveals the distribution of citing and cited journals in TLS research. On the left side, representing citing journals, prominent disciplines include Molecular/Biology/Immunology and Dentistry/Dermatology/Surgery, which primarily cite publications in Molecular Biology/Genetics journals (**Figure 7A**).

TLS-related articles have been published across 290 academic journals, with 109 journals contributing two or more articles. Leading the publication count are “Frontiers in Immunology” (119), “Cancers” (30), “Journal for Immunotherapy of Cancer” (28), “Oncoimmunology” (27), and “Nature Communications” (17) (**Table 5**). “Frontiers in Immunology” stands out as a central player in the network of journal associations (**Figure 7B**). However, the most cited journal in TLS research is “Nature” (cited 3230 times). The top three most highly cited articles on TLS were all published in “Nature.” For instance, Helmink BA et al.’s article titled “B cells and tertiary lymphoid structures promote immunotherapy response” has amassed 1186 citations (60).

**Table 5:** Top ten produced (left) journals and cited journals (right) related to the research for TLS research.

Rank	Source	Documents	IF-2023	Rank	Source	Citations	Total link strength
1	Frontiers in immunology	119	7.3	1	Nature	3230	646
2	Cancers	30	5.2	2	Frontiers in Immunology	2929	1539
3	Journal for Immunotherapy of Cancer	28	10.9	3	Oncoimmunology	1155	482
4	Oncoimmunology	27	7.2	4	Nature Medicine	1088	124
5	Nature Communications	17	16.6	5	Nature Reviews Immunology	1074	143
6	Frontiers in Oncology	15	4.7	6	Clinical Cancer Research	837	222
7	Cancer Immunology Immunotherapy	11	5.8	7	Cancer Immunology Research	812	190
8	Clinical Cancer Research	9	11.5	8	Nature Communications	811	187
9	Plos One	9	3.7	9	Cancer Research	808	244
10	PNAS	9	11.1	10	Journal for Immunotherapy of Cancer	708	316

This analysis illustrates a complex network of journal relationships in TLS research, highlighting key journals and influential articles that have shaped the discourse and advancements in the field.

## 4 Discussion

Our findings demonstrate a rapid growth in studies focusing on TLS over the past decade, driven by advancements in immunotherapy, particularly ICIs, which have revolutionized anti-tumor treatments. Concurrently, there is a burgeoning understanding of the tumor immune microenvironment, where TLS plays a pivotal role. Based on the results analyzed above, we will further summarize and discuss the research value of TLS in terms of significant advancements, current status, clinical applications, and challenges.

### 4.1 Significant advancements and current status

Autoimmune diseases are closely associated with dysregulated immunity, and the formation and maturation of TLS may play a significant role in this process. Furthermore, TLS in various immune disorders may exhibit distinct characteristic cell populations. For instance, Th17 cells are implicated in developing autoimmune encephalomyelitis within the central nervous system (61). Similarly, the enrichment of Th17 cells is closely linked to the progression of follicular pancreatitis (62). Other subtypes of peripheral helper T cells and follicular helper T cells also contribute to the formation and maturation of TLS in immune disease lesions, including rheumatoid arthritis, Sjögren syndrome, and systemic lupus erythematosus (63, 64). Additionally, other immune cell subsets within TLS, such as dendritic cells, B lymphocytes, and macrophages, have been shown to play diverse roles in the development and progression of autoimmune diseases (11, 65). Emerging technologies, such as single-cell sequencing, are increasingly being utilized to analyze cell subsets in TLS associated with autoimmune diseases (11, 66, 67). These advanced techniques may offer valuable insights for identifying a broader range of immune cell subsets and elucidating their mechanisms of action.

Advancements have also been made in understanding the mechanisms of action and predictive value of immune cell subsets in tumors. Early studies have indicated that B cell enrichment in TLS is associated with a favorable prognosis in various cancers, including lung cancer, pancreatic cancer, gastric cancer, and melanoma (49, 60, 68, 69). The application of single-cell sequencing, spatial transcriptomics, and immunohistochemistry aims to identify B cells with specific markers in TLS. For instance, TCL1A-expressing B cells have been linked to oral squamous cell carcinoma, distinct B cell subsets have been identified in nasopharyngeal carcinoma, an interferon-stimulated B cell subtype has been associated with muscle-invasive bladder cancer, and CD20+CD22+ADAM28+ ICI-responsive B cells have also been characterized (70–73).

An increasing number of T cell subtypes within TLS have been analyzed. For instance, TCF1/TCF7-positive T cells located in and around TLS are associated with a better prognosis for oral cancer (74). Chemokine (CXCL13)-high-expressing T cells are also believed to be critical for TLS maturation, thereby influencing tumor progression (75–77). Furthermore, different T cell subtypes within TLS may produce opposing anti-tumor effects (74, 78–80). Additionally, immune cells such as dendritic cells, macrophages, and natural killer cells within TLS play essential roles in shaping the tumor immune microenvironment (81, 82). These immune cells are also continuously being identified and evaluated (65, 83, 84).

Due to their small size, diverse shapes, and multifocal spatial distribution, TLS present a significant challenge for pathologists and physicians in determining their nature. Consequently, researchers are exploring additional markers beyond the commonly used ones, such as CD20, CD4, and CD8. For instance, CD23 expression, a TLS-specific marker, has been found to be positively correlated with disease-free survival and overall survival in breast cancer (85). L1CAM is also recognized as a reliable marker for mature TLS associated with endometrial cancer (86). Furthermore, BCL6-expressing B cells and CD21-positive follicular dendritic cells have been shown to be concentrated in TLS in ovarian cancer (87). Similarly, LGALS2 has been identified as a key marker within TLS in breast cancer, demonstrating a positive correlation with prolonged survival (84). It is likely that more markers will be revealed in the future.

Another point of interest is the relationship between TLS maturity and tumor progression. Several studies have demonstrated that mature TLS is positively correlated with the prognosis of solid tumors treated with ICIs (88–90). This correlation may be linked to the enrichment of immunologically activated cells within mature TLS (13, 91). In contrast, immature TLS appears to be enriched with immunosuppressive immune cells and exhibits immunosuppressive characteristics (32, 83, 92). Immature TLS is likely to contribute to resistance against ICIs (93). For instance, peritoneal metastases derived from gastric cancers are often enriched with tumor-infiltrating macrophages and regulatory T cells, while exhibiting a reduced presence of plasma cells, thereby creating an immunosuppressive microenvironment (83). It is likely that increasing differences between TLS in various cancer types and metastatic lesions will be identified and assessed.

### 4.2 Clinical applications

The search for reliable prognostic markers for immunotherapy has long been a focus of clinical research. However, dependable markers with broad applicability are still lacking in solid tumors (94, 95). TLS appears to be a promising candidate (96). Results from several clinical trials have demonstrated that TLS is one of the biomarkers associated with responses to neoadjuvant immunotherapy in conditions such as hepatocellular carcinoma, NSCLC, urothelial cancer, and esophageal squamous cell carcinoma (97–100). These findings suggest that effective ICIs treatment may promote TLS maturation, thereby creating a positive feedback loop. Furthermore, several studies have indicated a close association between chemotherapy and TLS, with chemotherapy also capable of inducing TLS maturation, which may enhance the efficacy of immunotherapy (101–104). The results of multiple clinical studies have shown that neoadjuvant immunochemotherapy combinations exhibit superior efficacy compared to either neoadjuvant chemotherapy or neoadjuvant immunotherapy alone (105–107). The maturity of TLS may be one of the influencing factors in this context. It is anticipated that effectively inducing intratumoral TLS maturation will become a focal point of research in the future.

### 4.3 Challenges of TLS

As previously mentioned, the predictive efficacy of mature TLS and immature TLS against tumors may be contradictory. Furthermore, TLS located in different regions of a tumor may exhibit distinct cellular compositions, resulting in variations in the immune microenvironment. These factors raise concerns about the reliability of using TLS as prognostic biomarkers, particularly given the inherent differences among various cancer types. Therefore, it may be beneficial to identify TLS with specific markers in particular cancers as predictive factors. However, due to the complexity and diversity of TLS structures, standardized diagnostic criteria remain lacking, despite the ongoing application of techniques such as immunohistochemistry, hematoxylin and eosin staining, and gene sequencing. The introduction of new diagnostic methods not only increases the costs associated with learning and treatment but may also hinder their widespread implementation. Lastly, the mechanisms underlying the transformation from immature TLS to mature TLS are not yet fully understood, and the distinctions between TLS in primary lesions and those in metastatic lesions are not completely recognized. These challenges may continue to motivate the scientific community to further investigate TLS, and we anticipate more positive outcomes in the future.

## 5 Limitation

The study still has a few limitations. First, the data associated with the TLS were sourced from a single database (the WOSCC) so that they would accommodate the data format for bibliometric tools in all “VOSviewer”, “CiteSpace”, and “bibliometrics”. This could have led to selection bias. There are other data sources, such as PubMed or Scopus, that can usually only be used effectively with one of the bibliometric tools (usually “VOSviewer”). To reduce selection bias, we used three bibliometric tools to conduct a comprehensive analysis. In addition, our study only included articles published in English, which may have led to the presence of language bias. In order to obtain a more comprehensive analysis, it might be more appropriate for future investigations to include publications in languages beyond English.

## 6 Conclusion

This study systematically reviewed global publications on TLS, analyzed their bibliometric characteristics, and identified influential articles. In summary, our bibliometric analysis has traced the evolution of TLS research and highlighted shifting research priorities over the past decade. These findings provide valuable insights into the pivotal role of TLS in immunotherapy and offer glimpses into future directions for this rapidly evolving field.

## Data Availability

The raw data supporting the conclusions of this article will be made available by the authors, without undue reservation.

## References

[1] Mao X Xu J Wang W Liang C Hua J Liu J. Crosstalk between cancer-associated fibroblasts and immune cells in the tumor microenvironment: new findings and future perspectives. Mol Cancer. (2021) 20:131. doi: 10.1186/s12943-021-01428-1 34635121 PMC8504100

[2] Brummel K Eerkens AL De Bruyn M Nijman HW. Tumour-infiltrating lymphocytes: from prognosis to treatment selection. Br J Cancer. (2023) 128:451–8. doi: 10.1038/s41416-022-02119-4 PMC9938191 36564565

[3] Bremnes RM Al-Shibli K Donnem T Sirera R Al-Saad S Andersen S. The role of tumor-infiltrating immune cells and chronic inflammation at the tumor site on cancer development, progression, and prognosis: emphasis on non-small cell lung cancer. J Thorac Oncol. (2011) 6:824–33. doi: 10.1097/JTO.0b013e3182037b76 21173711

[4] Wouters MCA Nelson BH. Prognostic significance of tumor-infiltrating B cells and plasma cells in human cancer. Clin Cancer Res. (2018) 24:6125–35. doi: 10.1158/1078-0432.Ccr-18-1481 30049748

[5] Sun Q Hong Z Zhang C Wang L Han Z Ma D. Immune checkpoint therapy for solid tumours: clinical dilemmas and future trends. Signal Transduct Target Ther. (2023) 8:320. doi: 10.1038/s41392-023-01522-4 37635168 PMC10460796

[6] Miller BC Sen DR Al Abosy R Bi K Virkud YV Lafleur MW. Subsets of exhausted CD8(+) T cells differentially mediate tumor control and respond to checkpoint blockade. Nat Immunol. (2019) 20:326–36. doi: 10.1038/s41590-019-0312-6 PMC6673650 30778252

[7] Kim CG Kim G Kim KH Park S Shin S Yeo D. Distinct exhaustion features of T lymphocytes shape the tumor-immune microenvironment with therapeutic implication in patients with non-small-cell lung cancer. J Immunother Cancer. (2021) 9:e002780. doi: 10.1136/jitc-2021-002780 34907028 PMC8671984

[8] Park S Ock CY Kim H Pereira S Park S Ma M. Artificial intelligence-powered spatial analysis of tumor-infiltrating lymphocytes as complementary biomarker for immune checkpoint inhibition in non-small-cell lung cancer. J Clin Oncol. (2022) 40:1916–28. doi: 10.1200/jco.21.02010 PMC9177249 35271299

[9] Lopez De Rodas M Nagineni V Ravi A Datar IJ Mino-Kenudson M Corredor G. Role of tumor infiltrating lymphocytes and spatial immune heterogeneity in sensitivity to PD-1 axis blockers in non-small cell lung cancer. J Immunother Cancer. (2022) 10:e004440. doi: 10.1136/jitc-2021-004440 35649657 PMC9161072

[10] Schumacher TN Thommen DS. Tertiary lymphoid structures in cancer. Science. (2022) 375:eabf9419. doi: 10.1126/science.abf9419 34990248

[11] Sato Y Jain A Ohtsuki S Okuyama H Sturmlechner I Takashima Y. Stem-like CD4(+) T cells in perivascular tertiary lymphoid structures sustain autoimmune vasculitis. Sci Transl Med. (2023) 15:eadh0380. doi: 10.1126/scitranslmed.adh0380 37672564 PMC11131576

[12] Sato Y Silina K Van Den Broek M Hirahara K Yanagita M. The roles of tertiary lymphoid structures in chronic diseases. Nat Rev Nephrol. (2023) 19:525–37. doi: 10.1038/s41581-023-00706-z PMC10092939 37046081

[13] Ling Y Zhong J Weng Z Lin G Liu C Pan C. The prognostic value and molecular properties of tertiary lymphoid structures in oesophageal squamous cell carcinoma. Clin Transl Med. (2022) 12:e1074. doi: 10.1002/ctm2.1074 36245289 PMC9574489

[14] Fridman WH Meylan M Petitprez F Sun C M Italiano A Sautès-Fridman C. B cells and tertiary lymphoid structures as determinants of tumour immune contexture and clinical outcome. Nat Rev Clin Oncol. (2022) 19:441–57. doi: 10.1038/s41571-022-00619-z 35365796

[15] Mo Z Liu J Zhang J Deng Y Xu M Jiang Y. Association of NRAS mutations and tertiary lymphoid structure formation with clinical outcomes of adjuvant PD-1 inhibitors for acral melanoma. Int Immunopharmacol. (2023) 124:110973. doi: 10.1016/j.intimp.2023.110973 37769536

[16] Masuda T Tanaka N Takamatsu K Hakozaki K Takahashi R Anno T. Unique characteristics of tertiary lymphoid structures in kidney clear cell carcinoma: prognostic outcome and comparison with bladder cancer. J Immunother Cancer. (2022) 10:e003883. doi: 10.1136/jitc-2021-003883 35314433 PMC8938705

[17] Zhu S Liu Y Gu Z Zhao Y. Research trends in biomedical applications of two-dimensional nanomaterials over the last decade - A bibliometric analysis. Adv Drug Delivery Rev. (2022) 188:114420. doi: 10.1016/j.addr.2022.114420 35835354

[18] Jiang S Liu Y Zheng H Zhang L Zhao H Sang X. Evolutionary patterns and research frontiers in neoadjuvant immunotherapy: a bibliometric analysis. Int J Surg. (2023) 109:2774–83. doi: 10.1097/js9.0000000000000492 PMC10498839 37216225

[19] Pei Z Chen S Ding L Liu J Cui X Li F. Current perspectives and trend of nanomedicine in cancer: A review and bibliometric analysis. J Control Release. (2022) 352:211–41. doi: 10.1016/j.jconrel.2022.10.023 36270513

[20] Yang S Hao S Ye H Zhang X. Global research on the crosstalk between intestinal microbiome and colorectal cancer: A visualization analysis. Front Cell Infect Microbiol. (2023) 13:1083987. doi: 10.3389/fcimb.2023.1083987 37009513 PMC10050574

[21] Rondanelli M Perna S Peroni G Guido D. A bibliometric study of scientific literature in Scopus on botanicals for treatment of androgenetic alopecia. J Cosmet Dermatol. (2016) 15:120–30. doi: 10.1111/jocd.12198 26608588

[22] Di Cosmo A Pinelli C Scandurra A Aria M D'aniello B. Research trends in octopus biological studies. Anim (Basel). (2021) 11:1808. doi: 10.3390/ani11061808 PMC8233767 34204419

[23] Zhang C Wang XY Zuo JL Wang XF Feng XW Zhang B. Localization and density of tertiary lymphoid structures associate with molecular subtype and clinical outcome in colorectal cancer liver metastases. J Immunother Cancer. (2023) 11:e006425. doi: 10.1136/jitc-2022-006425 36759015 PMC9923349

[24] Oba M Nakanishi Y Amano T Okamura K Tsuchikawa T Nakamura T. Stratification of postoperative prognosis by invasive tumor thickness in perihilar cholangiocarcinoma. Ann Surg Oncol. (2021) 28:2001–9. doi: 10.1245/s10434-020-09135-9 33040247

[25] Hou Y Qiao S Li M Han X Wei X Pang Y. The gene signature of tertiary lymphoid structures within ovarian cancer predicts the prognosis and immunotherapy benefit. Front Genet. (2022) 13:1090640. doi: 10.3389/fgene.2022.1090640 36704336 PMC9871364

[26] Rakaee M Kilvaer TK Jamaly S Berg T Paulsen EE Berglund M. Tertiary lymphoid structure score: a promising approach to refine the TNM staging in resected non-small cell lung cancer. Br J Cancer. (2021) 124:1680–9. doi: 10.1038/s41416-021-01307-y PMC8110789 33723388

[27] Lynch KT Young SJ Meneveau MO Wages NA Engelhard VH Slingluff CL Jr. Heterogeneity in tertiary lymphoid structure B-cells correlates with patient survival in metastatic melanoma. J Immunother Cancer. (2021) 9:e002273. doi: 10.1136/jitc-2020-002273 34103353 PMC8190052

[28] Liu Z Meng X Tang X Zou W He Y. Intratumoral tertiary lymphoid structures promote patient survival and immunotherapy response in head neck squamous cell carcinoma. Cancer Immunol Immunother. (2023) 72:1505–21. doi: 10.1007/s00262-022-03310-5 PMC10198854 36481914

[29] Cabrita R Lauss M Sanna A Donia M Skaarup Larsen M Mitra S. Tertiary lymphoid structures improve immunotherapy and survival in melanoma. Nature. (2020) 577:561–5. doi: 10.1038/s41586-019-1914-8 31942071

[30] Hu L Li X Yang C Zhou B Du C Jiang N. Prognostic value of tertiary lymphoid structures in hepatocellular carcinoma: a meta-analysis and systematic review. Front Immunol. (2024) 15:1390938. doi: 10.3389/fimmu.2024.1390938 38887293 PMC11180782

[31] Wang YQ Chen WJ Zhou W Dong KQ Zuo L Xu D. Integrated analysis of tertiary lymphoid structures and immune infiltration in ccRCC microenvironment revealed their clinical significances: a multicenter cohort study. J Immunother Cancer. (2024) 12:e008613. doi: 10.1136/jitc-2023-008613 38908856 PMC11331356

[32] Xu W Lu J Liu WR Anwaier A Wu Y Tian X. Heterogeneity in tertiary lymphoid structures predicts distinct prognosis and immune microenvironment characterizations of clear cell renal cell carcinoma. J Immunother Cancer. (2023) 11:e006667. doi: 10.1136/jitc-2023-006667 38040418 PMC10693897

[33] Asrir A Tardiveau C Coudert J Laffont R Blanchard L Bellard E. Tumor-associated high endothelial venules mediate lymphocyte entry into tumors and predict response to PD-1 plus CTLA-4 combination immunotherapy. Cancer Cell. (2022) 40:318–334.e9. doi: 10.1016/j.ccell.2022.01.002 35120598

[34] Zhan Z Shi-Jin L Yi-Ran Z Zhi-Long L Xiao-Xu Z Hui D. High endothelial venules proportion in tertiary lymphoid structure is a prognostic marker and correlated with anti-tumor immune microenvironment in colorectal cancer. Ann Med. (2023) 55:114–26. doi: 10.1080/07853890.2022.2153911 PMC9754014 36503344

[35] Vella G Guelfi S Bergers G. High endothelial venules: A vascular perspective on tertiary lymphoid structures in cancer. Front Immunol. (2021) 12:736670. doi: 10.3389/fimmu.2021.736670 34484246 PMC8416033

[36] Song IH Heo SH Bang WS Park HS Park IA Kim YA. Predictive value of tertiary lymphoid structures assessed by high endothelial venule counts in the neoadjuvant setting of triple-negative breast cancer. Cancer Res Treat. (2017) 49:399–407. doi: 10.4143/crt.2016.215 27488875 PMC5398384

[37] Sawada J Hiraoka N Qi R Jiang L Fournier-Goss AE Yoshida M. Molecular signature of tumor-associated high endothelial venules that can predict breast cancer survival. Cancer Immunol Res. (2022) 10:468–81. doi: 10.1158/2326-6066.Cir-21-0369 PMC8976767 35201289

[38] Wang B Han Y Liu J Zhang X Deng Y Jiang Y. Intratumoral high endothelial venules in solid tumors: a pooled study. Front Immunol. (2024) 15:1401118. doi: 10.3389/fimmu.2024.1401118 39040120 PMC11260642

[39] Luo J Shi X Liu Y Wang J Wang H Yang X. Immune checkpoint ligands expressed on mature high endothelial venules predict poor prognosis of NSCLC: have a relationship with CD8(+) T lymphocytes infiltration. Front Immunol. (2024) 15:1302761. doi: 10.3389/fimmu.2024.1302761 38390332 PMC10882939

[40] Ye D Jin Y Weng Y Cui X Wang J Peng M. High endothelial venules predict response to PD-1 inhibitors combined with anti-angiogenesis therapy in NSCLC. Sci Rep. (2023) 13:16468. doi: 10.1038/s41598-023-43122-w 37777573 PMC10543372

[41] Chelvanambi M Fecek RJ Taylor JL Storkus WJ. STING agonist-based treatment promotes vascular normalization and tertiary lymphoid structure formation in the therapeutic melanoma microenvironment. J Immunother Cancer. (2021) 9:e001906. doi: 10.1136/jitc-2020-001906 33526609 PMC7852948

[42] Johansson-Percival A He B Li ZJ Kjellén A Russell K Li J. *De novo* induction of intratumoral lymphoid structures and vessel normalization enhances immunotherapy in resistant tumors. Nat Immunol. (2017) 18:1207–17. doi: 10.1038/ni.3836 28892469

[43] Xu Y Li Z Yang Y Zhang Y Li L Zhou Y. Association between MRI radiomics and intratumoral tertiary lymphoid structures in intrahepatic cholangiocarcinoma and its prognostic significance. J Magn Reson Imaging. (2024) 60:715–28. doi: 10.1002/jmri.29128 37942838

[44] Xu Y Li Z Yang Y Li L Zhou Y Ouyang J. A CT-based radiomics approach to predict intra-tumoral tertiary lymphoid structures and recurrence of intrahepatic cholangiocarcinoma. Insights Imaging. (2023) 14:173. doi: 10.1186/s13244-023-01527-1 37840098 PMC10577112

[45] Li K Ji J Li S Yang M Che Y Xu Z. Analysis of the correlation and prognostic significance of tertiary lymphoid structures in breast cancer: A radiomics-clinical integration approach. J Magn Reson Imaging. (2024) 59:1206–17. doi: 10.1002/jmri.28900 37526043

[46] Astorri E Scrivo R Bombardieri M Picarelli G Pecorella I Porzia A. CX3CL1 and CX3CR1 expression in tertiary lymphoid structures in salivary gland infiltrates: fractalkine contribution to lymphoid neogenesis in Sjogren’s syndrome. Rheumatol (Oxford). (2014) 53:611–20. doi: 10.1093/rheumatology/ket401 24324211

[47] Corsiero E Nerviani A Bombardieri M Pitzalis C. Ectopic lymphoid structures: powerhouse of autoimmunity. Front Immunol. (2016) 7:430. doi: 10.3389/fimmu.2016.00430 27799933 PMC5066320

[48] Gu-Trantien C Loi S Garaud S Equeter C Libin M De Wind A. CD4^+^ follicular helper T cell infiltration predicts breast cancer survival. J Clin Invest. (2013) 123:2873–92. doi: 10.1172/jci67428 PMC3696556 23778140

[49] Germain C Gnjatic S Tamzalit F Knockaert S Remark R Goc J. Presence of B cells in tertiary lymphoid structures is associated with a protective immunity in patients with lung cancer. Am J Respir Crit Care Med. (2014) 189:832–44. doi: 10.1164/rccm.201309-1611OC 24484236

[50] Sautès-Fridman C Lawand M Giraldo NA Kaplon H Germain C Fridman WH. Tertiary lymphoid structures in cancers: prognostic value, regulation, and manipulation for therapeutic intervention. Front Immunol. (2016) 7:407. doi: 10.3389/fimmu.2016.00407 27752258 PMC5046074

[51] Pinter M Scheiner B Pinato DJ. Immune checkpoint inhibitors in hepatocellular carcinoma: emerging challenges in clinical practice. Lancet Gastroenterol Hepatol. (2023) 8:760–70. doi: 10.1016/s2468-1253(23)00147-4 37327807

[52] Calderaro J Petitprez F Becht E Laurent A Hirsch TZ Rousseau B. Intra-tumoral tertiary lymphoid structures are associated with a low risk of early recurrence of hepatocellular carcinoma. J Hepatol. (2019) 70:58–65. doi: 10.1016/j.jhep.2018.09.003 30213589

[53] Li J Zhang L Xing H Geng Y Lv S Luo X. The absence of intra-tumoral tertiary lymphoid structures is associated with a worse prognosis and mTOR signaling activation in hepatocellular carcinoma with liver transplantation: A multicenter retrospective study. Adv Sci (Weinh). (2024) 11:e2309348. doi: 10.1002/advs.202309348 38498682 PMC11151010

[54] Dieu-Nosjean MC Goc J Giraldo NA Sautès-Fridman C Fridman WH. Tertiary lymphoid structures in cancer and beyond. Trends Immunol. (2014) 35:571–80. doi: 10.1016/j.it.2014.09.006 25443495

[55] Dieu-Nosjean MC Giraldo NA Kaplon H Germain C Fridman WH Sautès-Fridman C. Tertiary lymphoid structures, drivers of the anti-tumor responses in human cancers. Immunol Rev. (2016) 271:260–75. doi: 10.1111/imr.12405 27088920

[56] Noël G Fontsa ML Garaud S De Silva P De Wind A Van Den Eynden GG. Functional Th1-oriented T follicular helper cells that infiltrate human breast cancer promote effective adaptive immunity. J Clin Invest. (2021) 131:e139905. doi: 10.1172/jci139905 34411002 PMC8483751

[57] Buisseret L Garaud S De Wind A Van Den Eynden G Boisson A Solinas C. Tumor-infiltrating lymphocyte composition, organization and PD-1/PD-L1 expression are linked in breast cancer. Oncoimmunology. (2017) 6:e1257452. doi: 10.1080/2162402x.2016.1257452 28197375 PMC5283629

[58] Ramachandran M Vaccaro A Van De Walle T Georganaki M Lugano R Vemuri K. Tailoring vascular phenotype through AAV therapy promotes anti-tumor immunity in glioma. Cancer Cell. (2023) 41:1134–1151.e10. doi: 10.1016/j.ccell.2023.04.010 37172581

[59] Van Hooren L Vaccaro A Ramachandran M Vazaios K Libard S Van De Walle T. Agonistic CD40 therapy induces tertiary lymphoid structures but impairs responses to checkpoint blockade in glioma. Nat Commun. (2021) 12:4127. doi: 10.1038/s41467-021-24347-7 34226552 PMC8257767

[60] Helmink BA Reddy SM Gao J Zhang S Basar R Thakur R. B cells and tertiary lymphoid structures promote immunotherapy response. Nature. (2020) 577:549–55. doi: 10.1038/s41586-019-1922-8 PMC8762581 31942075

[61] Peters A Pitcher LA Sullivan JM Mitsdoerffer M Acton SE Franz B. Th17 cells induce ectopic lymphoid follicles in central nervous system tissue inflammation. Immunity. (2011) 35:986–96. doi: 10.1016/j.immuni.2011.10.015 PMC3422678 22177922

[62] Ryota H Ishida M Satoi S Yanagimoto H Yamamoto T Kosaka H. Clinicopathological and immunological features of follicular pancreatitis - a distinct disease entity characterised by Th17 activation. Histopathology. (2019) 74:709–17. doi: 10.1111/his.13802 30515871

[63] Huang Y Ba X Han L Wang H Lin W Chen Z. T peripheral helper cells in autoimmune diseases: What do we know? Front Immunol. (2023) 14:1145573. doi: 10.3389/fimmu.2023.1145573 37077922 PMC10106688

[64] Fonseca VR Romão VC Agua-Doce A Santos M López-Presa D Ferreira AC. The ratio of blood T follicular regulatory cells to T follicular helper cells marks ectopic lymphoid structure formation while activated follicular helper T cells indicate disease activity in primary sjögren’s syndrome. Arthritis Rheumatol. (2018) 70:774–84. doi: 10.1002/art.40424 29361207

[65] Zhao L Jin S Wang S Zhang Z Wang X Chen Z. Tertiary lymphoid structures in diseases: immune mechanisms and therapeutic advances. Signal Transduct Target Ther. (2024) 9:225. doi: 10.1038/s41392-024-01947-5 39198425 PMC11358547

[66] Han D Lee AY Kim T Choi JY Cho MY Song A. Microenvironmental network of clonal CXCL13+CD4+ T cells and Tregs in pemphigus chronic blisters. J Clin Invest. (2023) 133:e166357. doi: 10.1172/jci166357 37815865 PMC10688981

[67] Georgieva T Diddens J Friedrich V Lepennetier G Brand RM Lehmann-Horn K. Single-cell profiling indicates a high similarity between immune cells in the cerebrospinal fluid and in meningeal ectopic lymphoid tissue in experimental autoimmune encephalomyelitis. Front Immunol. (2024) 15:1400641. doi: 10.3389/fimmu.2024.1400641 38933267 PMC11199773

[68] Hiraoka N Ino Y Yamazaki-Itoh R Kanai Y Kosuge T Shimada K. Intratumoral tertiary lymphoid organ is a favourable prognosticator in patients with pancreatic cancer. Br J Cancer. (2015) 112:1782–90. doi: 10.1038/bjc.2015.145 PMC4647237 25942397

[69] Hennequin A Derangère V Boidot R Apetoh L Vincent J Orry D. Tumor infiltration by Tbet+ effector T cells and CD20+ B cells is associated with survival in gastric cancer patients. Oncoimmunology. (2016) 5:e1054598. doi: 10.1080/2162402x.2015.1054598 27057426 PMC4801425

[70] Xie W Lu J Chen Y Wang X Lu H Li Q. TCL1A-expressing B cells are critical for tertiary lymphoid structure formation and the prognosis of oral squamous cell carcinoma. J Transl Med. (2024) 22:477. doi: 10.1186/s12967-024-05292-7 38764038 PMC11103841

[71] Chen C Zhang Y Wu X Shen J. The role of tertiary lymphoid structure and B cells in nasopharyngeal carcinoma: Based on bioinformatics and experimental verification. Transl Oncol. (2024) 41:101885. doi: 10.1016/j.tranon.2024.101885 38295746 PMC10846412

[72] Yuan H Mao X Yan Y Huang R Zhang Q Zeng Y. Single-cell sequencing reveals the heterogeneity of B cells and tertiary lymphoid structures in muscle-invasive bladder cancer. J Transl Med. (2024) 22:48. doi: 10.1186/s12967-024-04860-1 38216927 PMC10787393

[73] Wu Z Zhou J Xiao Y Ming J Zhou J Dong F. CD20(+)CD22(+)ADAM28(+) B cells in tertiary lymphoid structures promote immunotherapy response. Front Immunol. (2022) 13:865596. doi: 10.3389/fimmu.2022.865596 35634306 PMC9130862

[74] Peng Y Xiao L Rong H Ou Z Cai T Liu N. Single-cell profiling of tumor-infiltrating TCF1/TCF7(+) T cells reveals a T lymphocyte subset associated with tertiary lymphoid structures/organs and a superior prognosis in oral cancer. Oral Oncol. (2021) 119:105348. doi: 10.1016/j.oraloncology.2021.105348 34044317

[75] Liu W You W Lan Z Ren Y Gao S Li S. An immune cell map of human lung adenocarcinoma development reveals an anti-tumoral role of the Tfh-dependent tertiary lymphoid structure. Cell Rep Med. (2024) 5:101448. doi: 10.1016/j.xcrm.2024.101448 38458196 PMC10983046

[76] Zhang D Jiang D Jiang L Ma J Wang X Xu X. HLA-A(+) tertiary lymphoid structures with reactivated tumor infiltrating lymphocytes are associated with a positive immunotherapy response in esophageal squamous cell carcinoma. Br J Cancer. (2024) 131:184–95. doi: 10.1038/s41416-024-02712-9 PMC11231239 38762674

[77] Liu Y Ye SY He S Chi DM Wang XZ Wen YF. Single-cell and spatial transcriptome analyses reveal tertiary lymphoid structures linked to tumour progression and immunotherapy response in nasopharyngeal carcinoma. Nat Commun. (2024) 15:7713. doi: 10.1038/s41467-024-52153-4 39231979 PMC11375053

[78] Joshi NS Akama-Garren EH Lu Y Lee DY Chang GP Li A. Regulatory T cells in tumor-associated tertiary lymphoid structures suppress anti-tumor T cell responses. Immunity. (2015) 43:579–90. doi: 10.1016/j.immuni.2015.08.006 PMC4826619 26341400

[79] Devi-Marulkar P Fastenackels S Karapentiantz P Goc J Germain C Kaplon H. Regulatory T cells infiltrate the tumor-induced tertiary lymphoïd structures and are associated with poor clinical outcome in NSCLC. Commun Biol. (2022) 5:1416. doi: 10.1038/s42003-022-04356-y 36566320 PMC9789959

[80] Hu C You W Kong D Huang Y Lu J Zhao M. Tertiary lymphoid structure-associated B cells enhance CXCL13(+)CD103(+)CD8(+) tissue-resident memory T-cell response to programmed cell death protein 1 blockade in cancer immunotherapy. Gastroenterology. (2024) 166:1069–84. doi: 10.1053/j.gastro.2023.10.022 38445519

[81] Posch F Silina K Leibl S Mündlein A Moch H Siebenhüner A. Maturation of tertiary lymphoid structures and recurrence of stage II and III colorectal cancer. Oncoimmunology. (2018) 7:e1378844. doi: 10.1080/2162402x.2017.1378844 29416939 PMC5798199

[82] Bugatti M Bergamini M Missale F Monti M Ardighieri L Pezzali I. A population of TIM4+FOLR2+ Macrophages localized in tertiary lymphoid structures correlates to an active immune infiltrate across several cancer types. Cancer Immunol Res. (2022) 10:1340–53. doi: 10.1158/2326-6066.Cir-22-0271 36122412

[83] Groen-Van Schooten TS Franco Fernandez R Van Grieken NCT Bos EN Seidel J Saris J. Mapping the complexity and diversity of tertiary lymphoid structures in primary and peritoneal metastatic gastric cancer. J Immunother Cancer. (2024) 12:e009243. doi: 10.1136/jitc-2024-009243 38955417 PMC11218001

[84] Li S Zhang N Zhang H Yang Z Cheng Q Wei K. Deciphering the role of LGALS2: insights into tertiary lymphoid structure-associated dendritic cell activation and immunotherapeutic potential in breast cancer patients. Mol Cancer. (2024) 23:216. doi: 10.1186/s12943-024-02126-4 39350165 PMC11441145

[85] Wang Q Sun K Liu R Song Y Lv Y Bi P. Single-cell transcriptome sequencing of B-cell heterogeneity and tertiary lymphoid structure predicts breast cancer prognosis and neoadjuvant therapy efficacy. Clin Transl Med. (2023) 13:e1346. doi: 10.1002/ctm2.1346 37525587 PMC10390819

[86] Horeweg N Workel HH Loiero D Church DN Vermij L Léon-Castillo A. Tertiary lymphoid structures critical for prognosis in endometrial cancer patients. Nat Commun. (2022) 13:1373. doi: 10.1038/s41467-022-29040-x 35296668 PMC8927106

[87] Al-Diwani A Theorell J Damato V Bull J Mcglashan N Green E. Cervical lymph nodes and ovarian teratomas as germinal centres in NMDA receptor-antibody encephalitis. Brain. (2022) 145:2742–54. doi: 10.1093/brain/awac088 PMC9486890 35680425

[88] Vanhersecke L Brunet M Guégan JP Rey C Bougouin A Cousin S. Mature tertiary lymphoid structures predict immune checkpoint inhibitor efficacy in solid tumors independently of PD-L1 expression. Nat Cancer. (2021) 2:794–802. doi: 10.1038/s43018-021-00232-6 35118423 PMC8809887

[89] Kinker GS Vitiello G Diniz AB Cabral-Piccin MP Pereira PHB Carvalho MLR. Mature tertiary lymphoid structures are key niches of tumour-specific immune responses in pancreatic ductal adenocarcinomas. Gut. (2023) 72:1927–41. doi: 10.1136/gutjnl-2022-328697 37230755

[90] Hayashi Y Makino T Sato E Ohshima K Nogi Y Kanemura T. Density and maturity of peritumoral tertiary lymphoid structures in oesophageal squamous cell carcinoma predicts patient survival and response to immune checkpoint inhibitors. Br J Cancer. (2023) 128:2175–85. doi: 10.1038/s41416-023-02235-9 PMC10241865 37016103

[91] Shu DH Ho WJ Kagohara LT Girgis A Shin SM Danilova L. Immune landscape of tertiary lymphoid structures in hepatocellular carcinoma (HCC) treated with neoadjuvant immune checkpoint blockade. bioRxiv. (2023) 26:2023. doi: 10.1101/2023.10.16.562104

[92] Fridman WH Sibéril S Pupier G Soussan S Sautès-Fridman C. Activation of B cells in Tertiary Lymphoid Structures in cancer: Anti-tumor or anti-self? Semin Immunol. (2023) 65:101703. doi: 10.1016/j.smim.2022.101703 36481358

[93] Ning J Hao J Guo F Hou X Li L Wang J. ABCB11 accumulated in immature tertiary lymphoid structures participates in xenobiotic metabolic process and predicts resistance to PD-1/PD-L1 inhibitors in head and neck squamous cell carcinoma. Transl Oncol. (2023) 36:101747. doi: 10.1016/j.tranon.2023.101747 37517143 PMC10407442

[94] Greten TF Villanueva A Korangy F Ruf B Yarchoan M Ma L. Biomarkers for immunotherapy of hepatocellular carcinoma. Nat Rev Clin Oncol. (2023) 20:780–98. doi: 10.1038/s41571-023-00816-4 37726418

[95] Ushio R Murakami S Saito H. Predictive markers for immune checkpoint inhibitors in non-small cell lung cancer. J Clin Med. (2022) 11:1855. doi: 10.3390/jcm11071855 35407463 PMC9000007

[96] Munoz-Erazo L Rhodes JL Marion VC Kemp RA. Tertiary lymphoid structures in cancer - considerations for patient prognosis. Cell Mol Immunol. (2020) 17:570–5. doi: 10.1038/s41423-020-0457-0 PMC7264315 32415259

[97] Ho WJ Zhu Q Durham J Popovic A Xavier S Leatherman J. Neoadjuvant cabozantinib and nivolumab converts locally advanced HCC into resectable disease with enhanced antitumor immunity. Nat Cancer. (2021) 2:891–903. doi: 10.1038/s43018-021-00234-4 34796337 PMC8594857

[98] Van Dijk N Gil-Jimenez A Silina K Hendricksen K Smit LA De Feijter JM. Preoperative ipilimumab plus nivolumab in locoregionally advanced urothelial cancer: the NABUCCO trial. Nat Med. (2020) 26:1839–44. doi: 10.1038/s41591-020-1085-z 33046870

[99] Cascone T Leung CH Weissferdt A Pataer A Carter BW Godoy MCB. Neoadjuvant chemotherapy plus nivolumab with or without ipilimumab in operable non-small cell lung cancer: the phase 2 platform NEOSTAR trial. Nat Med. (2023) 29:593–604. doi: 10.1038/s41591-022-02189-0 36928818 PMC10033402

[100] Wu C Zhang G Wang L Hu J Ju Z Tao H. Spatial proteomic profiling elucidates immune determinants of neoadjuvant chemo-immunotherapy in esophageal squamous cell carcinoma. Oncogene. (2024) 43:2751–67. doi: 10.1038/s41388-024-03123-z 39122893

[101] Delvecchio FR Fincham REA Spear S Clear A Roy-Luzarraga M Balkwill FR. Pancreatic cancer chemotherapy is potentiated by induction of tertiary lymphoid structures in mice. Cell Mol Gastroenterol Hepatol. (2021) 12:1543–65. doi: 10.1016/j.jcmgh.2021.06.023 PMC8529396 34252585

[102] Lanickova T Hensler M Kasikova L Vosahlikova S Angelidou A Pasulka J. Chemotherapy drives tertiary lymphoid structures that correlate with ICI-responsive TCF1+CD8+ T cells in metastatic ovarian cancer. Clin Cancer Res. (2024), OF1–OF17. doi: 10.1158/1078-0432.Ccr-24-1594 PMC11701433 39163092

[103] Lv J Wei Y Yin JH Chen YP Zhou GQ Wei C. The tumor immune microenvironment of nasopharyngeal carcinoma after gemcitabine plus cisplatin treatment. Nat Med. (2023) 29:1424–36. doi: 10.1038/s41591-023-02369-6 37280275

[104] Bertucci F De Nonneville A Finetti P Mamessier E. Predictive power of tertiary lymphoid structure signature for neoadjuvant chemotherapy response and immunotherapy benefit in HER2-negative breast cancer. Cancer Commun (Lond). (2023) 43:943–6. doi: 10.1002/cac2.12447 PMC10397558 37278484

[105] Chen S Yang Y Wang R Fang J. Neoadjuvant PD-1/PD-L1 inhibitors combined with chemotherapy had a higher ORR than mono-immunotherapy in untreated HNSCC: Meta-analysis. Oral Oncol. (2023) 145:106479. doi: 10.1016/j.oraloncology.2023.106479 37478574

[106] Sponholz S Koch A Mese M Becker S Sebastian M Fischer S. Lung cancer resection after immunochemotherapy versus chemotherapy in oligometastatic nonsmall cell lung cancer. Thorac Cardiovasc Surg. (2023) 71:656–63. doi: 10.1055/a-2028-7955 36746400

[107] Yu Z Liang C Xu Q Yuan Z Chen M Li R. The safety and efficacy of neoadjuvant PD-1 inhibitor plus chemotherapy for patients with locally advanced gastric cancer: A systematic review and meta-analysis. Int J Surg. (2024). Online ahead of print. doi: 10.1097/js9.0000000000002056 PMC11745722 39172720

